# Large River Effect or Frozen Kinetics: How Complex Nonlinear Living Systems Solve Optimization Problems

**DOI:** 10.1007/s11538-020-00766-3

**Published:** 2020-07-14

**Authors:** Alexander Filippov, Alexander Kovalev, Stanislav Gorb

**Affiliations:** 1 Functional Morphology and Biomechanics, Zoological Institute, Kiel University, 24118 Kiel, Germany; 2 Department of Electronic and Kinetic Properties of Non-linear Systems, Donetsk Institute for Physics and Engineering, National Academy of Science, 83114 Donetsk, Ukraine

**Keywords:** Evolution, Ecology, Adhesion, Frozen kinetics, Toy models

## Abstract

In this paper, we introduce general idea of trajectories attraction in phase space, which is very common phenomenon for the processes in the Nature. We start from a rather general biological example of natural selection, where adaptation to the environmental conditions can be described as attraction of some population distribution in the phenotype space to a center of ecological niche. The niche is mathematically represented as the “survival coefficient” which in turn can be linked to a kind of energy potential. This link between biological and physical approaches may be very useful for solution of a wide range of biological problems. In particular, we discuss an evolution in complex potential with a lot of valleys in a multi-dimensional space accompanied by the so-called large river effect, which corresponds to an extremely slow evolution of some, normally close to final, stages of the adaptation. This effect is related to the practically important states of the “frozen kinetics” which accompanies extremely wide spectrum of phenomena and allows understanding different physical and biological processes.

**Electronic supplementary material:**

The online version of this article (10.1007/s11538-020-00766-3) contains supplementary material, which is available to authorized users.

## Introduction

The mostly amazing feature of Nature is that it is extremely primitive and fantastically complex at the same time. It means that according to common belief, its basic laws are very simple or even naïve but observed structures and their dynamic behavior are unbelievably complicated. In some sense, this paper is devoted to a study of this strange combination. We will try to illustrate only some phenomena related to the realization of this quite common observation on a number of particular problems related to biological systems.

It is very difficult to imagine that all the physical motions and other more specific processes in the world are directly controlled by a “super-brain” which recalculates them for every particular step of their behavior. Thus, basic mechanisms are expected to be very simple and even primitive. It is well accepted now that it should be a kind of *self*-*organization*. For example, in biology general ideas of natural selection claim only that organisms basically transfer their genetically coded structure to the next generations with some deviations (mutations) and new forms being selected according to their advantages (or disadvantages) to survive and continue this transfer further. We will not speculate further about self-organization in philosophical sense and will concentrate on two main questions: (1) What does self-organization mean mathematically and (2) how one can apply this knowledge to generate new one?

## Biological Evolution from the Mathematical Point of View

Let us start from a very naïve first step and for definiteness select a biological example. Let us suppose that for some species, we have selected just one particular property which can be more or less definitely described quantitatively. As examples, one can imagine a length of legs, speed of running or any other parameter which can be directly measured. From the very beginning, it is quite obvious that there are some limitations of the value of any such parameter. It cannot be too large, or too small, and very likely has an optimum which is defined and limited by the conditions of living.

This clear assumption immediately leads to important mathematical and practical consequences. In particular, from mathematical point of view, it means that some function $S(x)$ exists in one-dimensional space $\left\{ x \right\}$ of the given parameter which is proportional to a probability to survive with a particular value $x$ of this parameter.

As a rule, we do not know the correct form of the function $S(x)$, but common belief is close to a supposition that this is a kind of normal (Gaussian) distribution around some optimal value $x_{0}$ corresponding to the maximal probability of survival with some width $w_{\text{S}}$ describing more or less acceptable deviations from this value where the probability sufficiently differs from zero:

1 $$ S\left( {x,x_{\text{S}} } \right) = { \exp }\left[ { - \left( {\frac{{x - x_{\text{S}} }}{{w_{\text{S}} }}} \right)^{2} } \right]. $$

However, instant distribution of the same parameter in a species also varies with some deviations. And probability to find its particular value at given time moment is proportional to some other function $F(x)$. Quite typical hypothesis is that this function is also a simple Gaussian. However, generally speaking it is not necessary and even if it is so, its center, width and amplitude do not coincide with the same values for the probability to survive $S(x,x_{\text{S}} )$.

To calculate a new generation, one has take into account that due to mutations, it also varies around every value $x \to x_{G}$ as around its new center $F(x) \to F(x_{G} )$. These deviations are defined by corresponding function $G\left( {x,x_{G} } \right) = { \exp }\left[ { - \left( {\frac{{x - x_{G} }}{{w_{G} }}} \right)^{2} } \right].$ Newly born generation is given by a product of the factor $G(x,x_{G} )$ and previous distribution $F(x_{G} )$ integrated over all the realizations of the parameter $x_{G}$: $F_{1} (x) = \int {{\text{d}}x_{G} } G(x,x_{G} )F(x_{G} )$. Finally, survived population is given by the product of all three functions:

2 $$ F_{2} (x,x_{\text{S}} ) = S(x,x_{\text{S}} )\int {{\text{d}}x_{G} } G(x,x_{G} )F(x_{G} ) $$

We simply multiplied three factors here: heredity, variability and selection. Even intuitively one can predict that at every iteration $k = 1,2, \ldots$, the survival coefficient $S(x,x_{\text{S}} )$ will cut off the parts of next population $F_{k + 1} (x,x_{\text{S}} )$, where $S(x,x_{\text{S}} )$ is small and support those ones which are closer to its center $x_{\text{S}}$. So, the distribution $F_{k + 1} (x,x_{\text{S}} )$ will be gradually attracted to the center.

This observation only seems to be very specific and made for a particular example taken from biological evolution. In fact, survival coefficient reflects an interaction between every individual $x$ of the array $F(x)$ with the environment. It only was written above for the definiteness and simplicity in form of collective mean-field factor $S(x,x_{\text{S}} )$.

Indeed, interaction with environment stimulates absolutely different objects of Nature to attract themselves to “their natural places” and, after all, stabilize them there. *Attraction* is the main key word here, and we applied this idea to the particular studies many times in our previous studies.

**Fig. 1:**
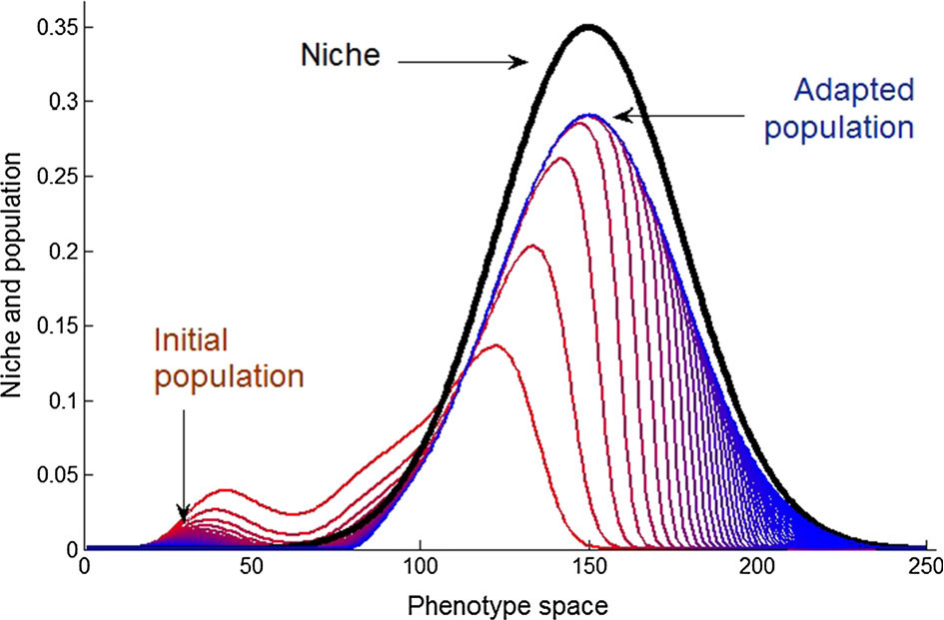
Attraction of the population to the “ecological niche.” The niche or “survival coefficient” is represented by the bold black line. Consequent stages of the evolution are shown by the curves with increasing amplitude of the second peak (the colors changing from red to blue). See also supplementary Movie_1_1 (Color figure online)

It is important to note that Nature is globally stable and majority of particular processes (and mathematical problems which we are solving) are stable as well. In very simple example discussed above, such stability was granted by the exponential decay of the Gaussian $S(x,x_{\text{S}} )$ which automatically suppressed large deviations of the parameter $x$ from $x_{\text{S}}$ (Fig. 1).

Of course, the Gaussian is a strong simplification of reality. However, based on its observation one can generate a very practical idea: More or less, it does not matter what happens in the central region of the attraction to moderate values of the parameters.

In fact, it is even not so important how many subsystems and interactions between them we include. The only feature we have to control is the system stability against large deviations from the center, where physically or biologically reasonable values of the parameters are expected. But, if the factors like $S(x,x_{\text{S}} )$ are chosen properly, the system will stabilize itself almost automatically (Filippov 1998, 1993).

Instead of “survival coefficient” $S(x,x_{\text{S}} )$, we would like to introduce at this point another, more physical concept. Even in “pure biology,” the probability to find particular realization of a variable $x$ is defined by an energy $U$ which is necessary to create a “niche” $U(x)$ for it. In physics, the coefficient analogous to $S(x,x_{\text{S}} )$ is produced by the so-called Boltzmann energy distribution:

3 $$ S\left( x \right) = { \exp }\left[ { - U\left( x \right)} \right] $$

where this energy also stays in the exponent (Landau and Lifshitz 1980; Lifshitz and Pitaevskii 1981).

**Fig. 2:**
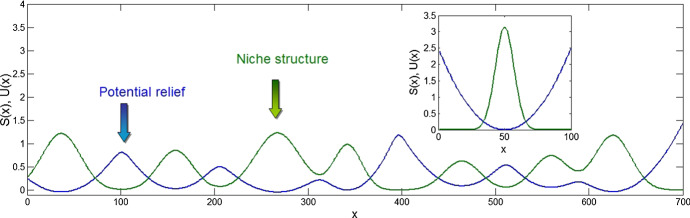
Relation between the niche structure (shown by green color) and effective potential relief (blue). Solitary niche (a peak) and corresponding potential valley are shown in the insert (Color figure online)

Independently on the branch of science, the energy tells about the system “what does it cost” to create a particular state. If we can find out or just estimate this cost from the experiments or observations, we can write down such energy as a potential relief $U(x)$ in space of parameters $\left\{ x \right\}$. It is important that we are not limited in the number of parameters in the space $\left\{ x \right\}$. If this number is large one, we will get a potential relief $U(x)$ in a multi-dimensional space only. It is difficult to imagine visually, but it is practically not a problem to calculate the survival coefficient in such space by the computer (Fig. 2).

Tending to minimize the energy, any system will relax to one or more minimums of the potential $U(x)$. If we know the correct $U(x)$, then our work is reduced to a simple task to observe how the system is doing this according to the equation of motion. In very simple and reduced manner, we can write it in the following form:

4 $$ \partial F(x,t)/\partial t = A\exp ( - U(x)) - F(x,t) $$

It is directly seen that with time, *t*, initial distribution of density $F(x,t = 0)$ will be “attracted” to the structure described by the structure of the potential:$F(x,t) \to A\exp ( - U(x))$.

## Attractor Approach

At this point, one could argue that such an approach to the various problems is a great oversimplification. In such an “approach,” we simply do not need any mathematics or simulation! We already know from the real experiments everything what we need and the calculation only masks the things which we already found without it.

Yes! And not! System really is attracted to the states which we somehow know in advance. But, the attraction is a process. Analyzing intermediate stages of the process, we can get a lot of knowledge which we would never get in another way. Let us mention some of the stages.

First of all, every process takes some time. If this time is long enough, we could never see the final static configuration. There are at least two reasons for this (second reason is discussed in the next section). The first one is that the characteristic time of the process is extremely long.

For example, all the galaxies develop and tend to some well predictable stages (like spherical or elliptic ones). But it takes billions of years. As result, we see many beautiful views of the spiral galaxies, including our own Milky Way. For us, they are visually “frozen” and all we can do is to compare different galaxies which we see at different stages of their evolution and make some conclusions about typical scenarios of their motion.

Fairly, everyday advantage of the proposed *attractor approach* is not so exotic as the study of the galaxies. Absolute majority of the things around us are actually seen at intermediate stages of their evolution. Even if formally they look static and we do not notice their evolution, e.g. stones are at an intermediate stage of their evolution, and they change only slowly with time. One can call this phenomenon “frozen kinetics”, but actually it is a particular realization of much more general phenomenon, so-called large river effect (Zumbach 1993; Bagnuls and Bervillier 1994; Filippov 1994, 1995).

**Fig. 3:**
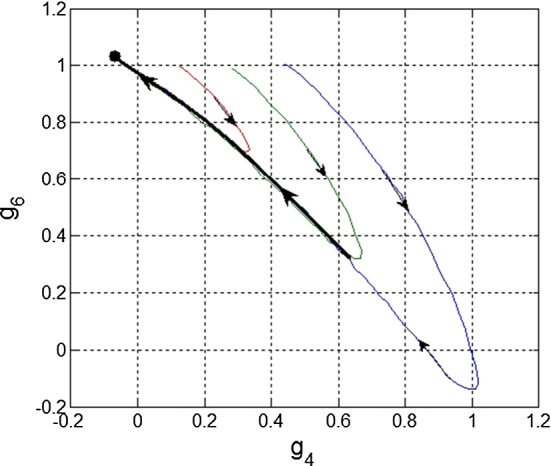
“Large river effect” in nonlinear differential equations. Particular flows with different initial conditions in phase space quickly tend to a common trajectory (shown by the bold black line) and slowly move along it to a fixed point

Accumulation of the small rivers into larger ones paradoxically repeats in much more sophisticated nonlinear dynamics, discussed above in the context of effective potential relief. It probably cannot be proven mathematically exactly, but quite common observation from very different scientific problems confirms that the same accumulation effect takes place for the phase trajectories of nonlinear equations generated by the effective potential relief (Fig. 3).

It is maybe not so important in the context of this particular discussion that many small rivers attract to a single one, but it is much more important here that due to typically very gentle slope, the river flows extremely slowly near to the river outlet.

## “Large River Effect” or “Frozen Kinetics”

Naïve belief of traditional (linear) mathematics was that the attraction to a fixed point $\vec{r}*$ with time $t \to \infty$ can be described by the exponential function $\left| {\vec{r} - \vec{r}^{*} } \right| = { \exp }\left( { - \alpha t} \right),$ where characteristic rate $\alpha$ can be relatively small, but still nonzero value. However, an experience obtained from the study of systems described by nonlinear realistic models tells us that in many case, such an attraction appears to be “logarithmically slow.”

Formally, it corresponds to variable exponent $\alpha$ which tends to zero $\alpha = \alpha (t) \to 0$ at $t \to \infty$. However, in research it usually means that in many cases, an evolution of system parameters becomes so slow that during the times typical for the problem, they practically do not change at all. That is why we can call this state *frozen kinetics*.

*Second reason* why we could never see the final static configuration (see above) is that all the systems are open to the external world. While they relax to the stationary state, something can happen in the larger world (in their environment) that partially or even completely changes the conditions at which the system started its evolution.

Mathematically, it means that we have to vary “static” potential relief as well. It becomes not static, but at least “quasi-static.” We obliged to change it: $U(x) \to U(x,t)$. Of course, only if we can make at least some guesses about its variation and compare later the results with the real observations. It completely modifies our research strategy. In many cases, we do not need the final, maybe dead, states of evolution, but we are much more interested in the intermediate long time living stages of it. Life is motion!

Sometimes, from a technical point of view, it can be not so convenient to vary the potential relief directly. In many cases, it is easier to substitute these modifications by investigating how external forces form and change an effective potential with time. If it is known, one can simply insert the forces into the right side of the equations of motion and solve modified version of the equation: $\partial F\left( {x,t} \right)/\partial t = A{ \exp }\left( { - U\left( x \right) - F\left( {x,t} \right)} \right) + \cdots {\text{External forces}}$.

## “Toy” Models and Their Significance

However, even if we do not know analytical form of such forces, let us remind that after all these forces are results of the interactions between the system under consideration and other ones, which were separated originally for a simplicity from the initial study and only because of these treated as the “external” systems.

So, in many cases it means that it is easier to include directly additional systems to the study from the very beginning. In some sense, this procedure returns the problem to its original form. However, it happens now in the parameters space of higher dimensionality.

By doing like this, in some sense we “oscillate” with the variants of approach. Les us remind that we started from as simple as possible presentation of the problem with the only one system moving in a potential valley and attracting to a final configuration inside it. As a rule, such oversimplification produces a caricature of much more complex reality. But, as with every good caricature, it allows us to see the essential features of the object, many of which we can even predict without numerical simulation.

It is very important after all that such a caricature gives us an image which we are able to easily keep in mind. We practically can see all important aspects: where an initial configuration starts, how it moves and how it is attracted to the probably unique or to very few valleys of the potential (Wilson and Kogut 1974; Ma 1976; Patashinskii and Pokrovskii 1979). It is good to have for beginning a very primitive model of the reality, which allows to estimate the model behavior in multi-parameter space at a realistic simulation time span. We normally call it “toy-model” and will use some of such models before making models more complex (realistic).

So, we would like to reproduce a “potential” in which system relaxes. It is often extremely difficult to formulate such normally multi-dimensional potential “a priori” or even imagine it. Fortunately, due to the increasing capability of computers we can do something new in this direction. We can perform numerical simulations with more or less intuitively understandable forces between the subsystems and in parallel to the simulation accumulate probability distribution of different measures. It gives us a posteriori representative and realistic *phase portrait* of the system.

Despite of the expected complexity and multi-dimensionality, this approach has a number of practical advantages. We can explore step by step its projections to different hyperplanes of parametric space and accumulate at least some static representation of the complex system dynamics. However, it is very easy to overestimate the capabilities of such approach because of, e.g., “human factor.”

Nature is generally simple, but complex from a combinatorial point of view. The best description of it is somewhere in a middle. We are confident that this approach opens for us a lot of freedom in an intuitive understanding of many different biological or related phenomena and even allows us to be braver in formulation and treatment of different models of biologically provoked mathematical models.

## Case Study from Ecology: Seed Dispersal by Ants

Let us mention as an example the following model of complex dynamic system which has been more extensively studied earlier (Gorb and Gorb 2003; Gorb et al. 2013). We are talking now about long-term ant-species-dependent dynamics of a myrmecochorous plant community. For this discussion, it is important that the ant species complex in the ecosystem is continuously changing in time and space, and the long-term effects of such ant–plant interactions on the plant community look quite difficult to study in standard experimental field approach.

Basic biological information about the system tells that seeds of myrmecochorous plants bear specialized lipid-rich appendages, elaiosomes, for attracting ants. Ant workers collect the seeds and usually carry them to their nests. From the point of view of our approach, it means that the space of the forest becomes non-uniform and forms a kind of the potential relief, where some positions are more preferable for the seeds to appear than other places. The origination of an effective “potential” is very complex and involves plenty of different factors. Let us mention only main of them.

Some seeds reach the nests, whereas others are dropped during transport. In the nests, the energy-laden elaiosomes are removed and consumed, whereas intact and viable seeds are commonly deposited either in underground nest chambers or in “waste piles” outside the nest. The ants benefit by receiving high quality food. In turn, myrmecochory provides plants with several selective advantages, such as protection of the seeds against predators and fire, avoidance of interspecific competition and reduction of the competition between the parental plant and its seedlings (Gorb and Gorb 2003).

It is important to note that for many plant species, seed dispersal by ants is the only dispersal method used. Seeds, depending on their dimensions and elaiosome size, are attractive to different ant species to a different extent and have different dropping rates during transport to/from the nest. In the frame of potential approach, it means that potential relief is different for the different species. But, the art of model construction consists on an appropriate choice of its complexity when it is already developed enough to account mostly important properties of the system and at the same time remain “somewhere in the middle.”

**Fig. 4:**
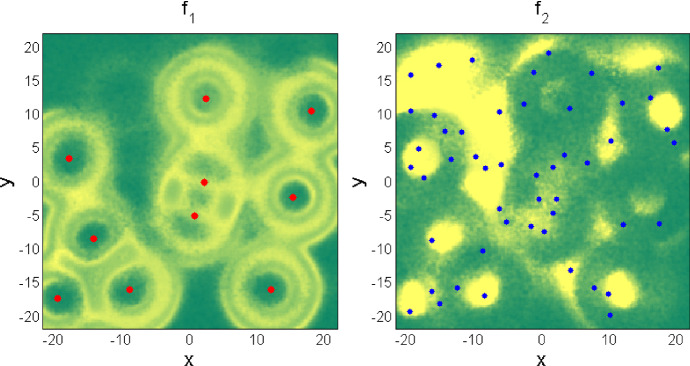
Compatibility between distributions of the anthills and seeds. The positions of two different kinds of anthills are show by the red (left subplot) and blue (right subplot) points, respectively. The densities $f_{1}$ and $f_{2}$ of different kinds of seeds are shown in two subplots by the color maps, where higher density corresponds to the brighter color (Color figure online)

By the way, this example is also favorable for an illustration of the general phenomenon of “frozen kinetics” (or, what is mathematically more correct, “large river effect”), because the ant species complex in the ecosystem is continuously changing in time and space, causing the long-term effects on ant–plant interactions and finally on the plant community (Fig. 4).

A distribution of the anthills in a particular forest is relatively stable. So, for a few years the seeds can “treat” caused by the anthills multi-valley effective potential as practically static one. Seed distributions and resulting distributions of plants are gradually attracted to some corresponding static densities caused by anthills. However, from time to time one or another ant family move and build the nest (anthill) at another site.

This movement catches density distribution of plants at some intermediate state, maybe close to the previously expected final static one, but still intermediate. From this moment, the system treats this already reached distribution as an initial condition, and the marathon of attraction to new potential minimums resumes. This process never finishes, at least while all components of the system, the forest and described species exist.

This observation completely changes a main goal of the study, which now is to determine a correlation between the number of nests of different ant species and the stability of the ecosystem under consideration, but not simple calculation of the final distribution. This example gives a very good base to illustrate also another idea, coming from physics and being extremely important for practical applications of the attractor approach. It is so-called *adiabatic approximation*.

## Adiabatic Approximation

For the first time, such an approximation was applied for calculation of the systems consisting of the electrons and ions (crystals, for example). The ions are much heavier and move much slower than electrons. In a very good approximation, one can treat their distribution in space (crystal lattice, for example) as a static one and study electrons moving in practically static potential created by the ions (Tolpygo 1950; Born and Huang 1954; Baryakhtar et al. 1999). However, positively charged ions repulse one another (by the way, anthills repulse one from another as well!) and need an attraction from the electrons to build stable crystal lattice.

The effective “adiabatic behaviour” is well pronounced in illustrative Movie_1_4. The description of the model generating this behavior is presented in (Filippov et al. 2000) where collective motion of the two groups of predator–prey pairs with essentially different mobilities is studied. It is seen directly that both fast and slow subgroups treat either instant or time-averaged configuration of another group (shown by small and big circles, respectively) as the effective potentials.

The analogy between system of ions and electrons on one side and anthills and seeds on the other side opens additional similarity between the approaches to physical and biological problems. It is alternative of using of continuous and discrete descriptions. In particular, depending on a physical problem, one can describe electrons as discrete particles moving in space or alternatively as continuous electron density, adapting to the discrete crystal lattice of ions. Each of the approaches has its own mathematical advantages and disadvantages and is actively used in physics for solving range of different problems.

The same freedom can be applied in biology, where we also can choose discrete or continual models depending on the situation or even combine both of them. For example, such combination was used by us for modeling seed–ant interactions. It is convenient to treat the distribution of seeds as continual density and construct effective potential for it starting from discrete set of the anthills.

In this case, we approach the problem as follows. A subsystem of ant nests is treated as two separate arrays of discrete points $\{ x_{kj} ,y_{kj} \}$. The nests are assumed to be randomly placed initially inside a fixed area in two-dimensional space $[x,y]$. Here, the indices $\{ k\}$ and $\{ j\}$ numerate two different species of ants $k = 1,2$ and a particular ant nest $j = 1, \ldots ,N_{k}$ in each set of ant nests.

Index $k = 1$ corresponds to the nest of larger ants having larger foraging territories, and $k = 2$ corresponds to smaller ants with smaller territories. Initial numbers of the nests at $t = 0$ are always fixed and equal to $N_{1} = 10$ and $N_{2} = 50$, respectively. For simplicity of simulations, we selected a square forest area of size $L_{x} \times L_{y}$ with constant equal sides $L_{x}$ and $L_{y}$: $L_{x} = L_{y} = 50$ meters. The initial positions of ant nests at $t = 0$ are given by the formulae: $x_{kj} = L_{x} \zeta_{kj}$, $y_{kj} = L_{y} \zeta_{kj}$, where $\zeta_{kj} (x,y)$ are $\delta$-correlated random numbers $< \zeta_{kj} \zeta_{k'j'} > = \delta_{kk'} \delta_{jj'}$ uniformly distributed in the interval $[ - 1/2 \div 1/2]$ along both spatial coordinates $x$ and $y$. Briefly, one can say that original nests are distributed uniformly and independently in the square of forest domain.

To construct effective potential for the seeds, or in other words to simulate the ability of the ants to disperse plant seeds, each ant nest is assumed to have, around its current position, a region with positive impact to the probability $z_{k}^{k'} (x,y)$ of plants to survive and produce new seeds for the next generation. Here, we use the subscripts $k = 1,2$ to numerate the nests of different ant species, and the superscripts $k' = 1,2$ to numerate different species of plants.

Large and small ants collect the seeds of different plant species having different sizes and dispose them either inside the rings near the borders of their territories (large ants) or inside small circles within the entire territory of the ant colony (small ants). In the model, it is accounted for in a different spatial structure of the *preference coefficients*:

5 $$ z_{1} (x,y) = \sum\limits_{k = 1}^{2} {B_{k}^{1} \sum\limits_{j = 1}^{{N_{1} }} {\exp \left[ { - \left( {\frac{{r - R_{1} }}{{R_{2} }}} \right)^{2} } \right]} } ;\quad z_{2} (x,y) = \sum\limits_{k = 1}^{2} {B_{k}^{2} \sum\limits_{j = 1}^{{N_{1} }} {\exp \left[ { - \left( {\frac{r}{{R_{2} }}} \right)^{2} } \right]} } $$

where $r = \sqrt {\left( {x - x_{kj} } \right)^{2} + \left( {y - y_{kj} } \right)^{2} }$ is the distance from an arbitrary point in area $L_{x} \times L_{y}$ to the position of the ant nest $\{ x_{kj} ,y_{kj} \}$. The values of the coefficients $B_{k}^{k'}$ should be chosen to reflect experimentally observed preferences in the choice of different seeds by different ant species.

The coefficients Eq. (5) form non-uniform surface which plays a role of the desired effective potential. Space–time evolution of the continual seed densities $f_{1,2} (x,y)$ is determined by their adaptation to this surface.

Namely, this approach we applied in original article (Gorb et al. 2013). However, it is obvious simplification and in principle, if it is needed for a particular study, one can refine the description further. Let us remind that the seeds are discrete objects in reality. They interact with the ants, and due to the activity of the ants, they move in space and as result attract to the preferable positions in the space described by the coefficients Eq. (5).

From the modeling aspect, the electrons are also attracted to the potential caused by the potential relief of the ions (or anthills) discretely distributed in some limited space (forest). If one imagines that electrons do not repulse, they should quickly collapse altogether into one or very few super-particles, collected in the mostly preferable place(s) of the lattice potential.

So, limited electron (seed) density is granted by the proper choice of the electron–electron interaction. This example illustrates two things: (1) close parallel between physical and biological study, and (2) importance of the correct choice of the interactions for the global stability of the model. In the presented study, we did not go deeply into the discussion of discrete nature of seeds and their mutual interactions and simply substituted them by a continuous density. However, in the cases when it is needed, one can do this. Below we will discuss such examples in detail.

## Continuous and Discrete Approaches in Modeling

Let us continue discussion of the advantages and disadvantages of the continuous and discrete approaches. It is very easy to represent or imagine one-dimensional distribution of some density. A little bit more sophisticated is to do the same for the two-dimensional surface. For a three-dimensional case, one could use *Fermi surface* representation: plot a projection of a surface of constant density (constant energy in Fermi surface case).

What to do, if dimensionality is equal to 4?

We can apply the same: visualize 3D surface of constant density for 3 of the parameters, as before, and represent on the video a flight along last of the parameter(s) in “next dimensionality.” In particular, this parameter can be time, but can be something else. Actually, in any plot we explore something moving along one of the properties. Why not to use the same idea? For example, we can simply rotate three-dimensional surfaces and watch.

The more parameters we use, the more continuous description seems to be less competitive in comparison to the discrete description. To the moment, we said almost nothing about long-range interaction. It was only briefly mentioned in discussion of adaptation of the seeds to adiabatically slow potential of anthills and electrons inside crystal lattice of slowly moving ions. In both cases, mutual interaction between the seeds and between the particles was repulsion. But, the seeds repulse only at very short distances by their hard cores which do not allow them to penetrate inside one another. Coulomb interaction between the electrons is the long-range one. Its potential $U_{\text{Coulomb}} \left( r \right) = 1/r$ very slowly decreases with the distance $r$. In the majority of the cases, the interaction between different substances is screened $U_{\text{Screen}} \left( r \right) = \exp \left( { - r/r_{0} } \right)/r$ at some characteristic distance $r_{0}$, but it is still going to zero not abrupt.

Let us imagine that we have only two continuous probability densities $\rho_{1} (r_{1} )$ and $\rho_{2} (r_{2} )$ (for simplicity, of course) interacting distantly. From mathematical point of view, it means that every point of the first density with coordinate $r_{1}$ interacts with each point $r_{2}$ of the second one with a long-range potential $U(r_{1} - r_{2} )$. It immediately causes non-local impact to the total energy of the system:

6 $$ U_{\text{interaction}} = \int {{\text{d}}r_{1} {\text{d}}r_{2} } \rho_{1} (r_{1} )U(r_{1} - r_{2} )\rho_{2} (r_{2} ) $$

This impact immediately complicates the equations of motion of the both densities:

7 $$ \partial \rho_{1} (r_{1} )/\partial t = \delta U_{\text{interaction}} /\delta r_{1} + \cdots = \int {{\text{d}}r_{2} } U(r_{1} - r_{2} )\rho_{2} (r_{2} ) + \cdots $$

and vice versa $\rho_{1} (r_{1} ) \leftrightarrow \rho_{2} (r_{2} )$. As result, in order to solve these equations numerically one has to integrate over complete volume, where both densities exist, at each step of the calculation! Let us remind now that in many cases, we deal with much larger number $N > > 2$ of the densities $\rho_{j} (r_{j} )$, where $j = 1,2, \ldots N$, and procedure becomes a nightmare. Why to use the continuous approach at all?

The answer is that there are many cases, where we do need long-range interactions to model particular system under consideration. Despite of the fact that in reality “everything interacts with everything” in many systems, long-range interactions can be combined preliminarily (before the simulations) into reduced combinations, where they compensate (screen) one another and allow us to limit the model by much simpler phenomenology with local interactions only: $U_{\text{interaction}} = \int {{\text{d}}r} \rho_{1} (r)U_{12} (r)\rho_{2} (r)$.

The observable world consists of the *phenomena*, and it is absolutely natural for us to operate on phenomenological level. Look around: We normally ignore the long-range interaction of electromagnetic waves with electronic density and simply accept that ray of light reflects from a surface in the local point $r$. Continuum media are given to us as obvious and that is it!

It is why we like such a description, when we can apply it. Nevertheless, there are some tasks, where we must take into account long-range interactions. If we cannot do this in continual approach, we should return to the discrete one or at least to a combination of both approaches.

Instead of Eqs. (6)–(7), let us write now direct interactions with a potential

8 $$ U_{\text{interact}} (r_{jk} ) = U(\left| {\vec{r}_{j} - \vec{r}_{k} } \right|) $$

depending on the absolute distance between the particles $\left| {\vec{r}_{j} - \vec{r}_{k} } \right|$ in the positions $\vec{r}_{j}$ and $\vec{r}_{k}$. Here, indexes $j$ and $k$ numerate $N$ particles of the system $j = 1,2, \ldots N$ and $k = 1,2, \ldots N$. Formally, the equation of motion is very simple:

9 $$ \partial \vec{r}_{j} /\partial t = - \sum\limits_{k} {\partial U_{\text{interact}} (\vec{r}_{jk} )/\partial \vec{r}_{j} } + F_{\text{external}} (\vec{r}_{j} ) $$

where $F_{\text{external}} (\vec{r}_{j} )$ is a sum of all the external forces, which are not included directly to the interaction $U_{\text{interact}} (r_{jk} )$. But, simplicity of this equation is illusory. Supplementary Movie_1_2 illustrates how complex can be a trajectory even in the case when the only one particle (red) pursues another one in relatively simple static potential relief. The trajectory complexity is explained by the following system properties.

First, the number of the operations at each time step grows with $N$ as $N \times N = N^{2}$, if each of $N$ particle interacts with all other particles. So, from the very beginning we have to think, how to minimize this number (maybe by means of restriction of the interactions by the properly defined close neighbors).

Second, in reduced form compact Eqs. (8)–(9) generate a giant amount of information about the system. To apply the equations practically, one has to specify spatial dependence of the interactions $U_{\text{interact}} (r_{jk} )$. For different kinds of particles in the same system, the interactions can be different. Besides, it is necessary to describe boundary conditions and directly introduce external interactions $F_{\text{external}} (\vec{r}_{j} )$. Complex behavior of such a system is illustrated in the Supplementary Movie_1_3 where some number of “predators” $N_{1}$ pursuing $N_{2}$ of “preys” is shown together with an effective “potential” caused by a redistribution of prey density $\rho (\vec{r})$ in the space.

Some of these forces can play an important role for the boundary conditions, and some of them may be entered to the equation as direct interactions with external subsystems, etc. Because of this uncertainty, we cannot continue moving further without specification of the particular system and model. In the framework of this paper, it is convenient to choose as example one of the problems studied below, which involves sufficient number of properties, to illustrate necessary aspects.

## Case Study from Biological Adhesion

Let us consider the adhesive system with multiple contacts which provide advantages in attachment of insects on rough substrates (Gorb 2001). We will not discuss here all the aspects of the problem, but concentrate directly on particular questions which are convenient to explore using basic ideas of our modeling approach.

It can be shown that thin tape-like contact tips (spatulae) of attachment hairs (setae) produce a maximal real contact area by application of shear force without slippage within the contact (Popov 2010). Due to this reason, the material flexibility is important for contact formation of adhesive pads. Flexible materials may generate large contact area between the pad and substrate at minimal normal load. On the other hand, elongated structures, made of very soft materials, have low mechanical stability: Insect setae made of very soft material can buckle and collapse resulting in so-called clusterization/condensation. Due to it, functional advantage from multiple adhesive contacts may strongly decrease.

From the mathematical point of view, we deal with two kinds of attraction. The first one is trivial attraction to a surface, and the second one is the attraction between the setae. So, it is typical problem, where internal and external interactions compete one with another and we cannot exclude any of them.

That is why, material properties of insect adhesive setae represent an optimization problem, which was solved in the course of biological evolution by the presence of gradients of thickness and mechanical properties along the setae. As result, the problem involves one more degree of freedom that makes the system even more reach example to be used here.

According to general ideology described above, we applied to this problem a minimalistic, but quite realistic model. We adapted the model to our particular problem by including gradient material properties to the modeled insect setae. The model includes the majority of the elements mentioned above in this introduction. Principally, it is constructed as follows: An array of initially parallel fibers is attached to a hard planar base. Stiffness of the fibers $K^{ \bot }$ is continuously varied along their length and can be changed from very soft one to much stiffer or even almost rigid one.

Since the discrete “nodes” of the array are ordered and during whole process belong to the same “fiber,” one can always define the nearest neighbors for each node. In some sense, it means that originally 3D multiple bodies problem can be partially (for the neighbors) transformed into effectively 1D and one-body problem.

This fantastic simplification practically does not reduce generality of problem. In some sense, it even makes model better for our goals, because it is the case, when good caricature of reality allows us better understand and imagine the system than the perfect but too overweighed complete image of reality.

Of course, some memory about 3D nature of the problem remains in this approximation as well. In particular, in this model it is reflected by the existence of both longitudinal $$ K^{\parallel } $$ and transversal $$ K^{ \bot } $$$$ \vec{F}_{j}^{ \bot } $$ stiffnesses of the fibers. Mathematically, they can be simulated by the following interaction between the segments $$ \vec{F}_{jk}^{||} = K^{||} (\vec{r}_{j} - \vec{r}_{k} )\left[ {1 - (\vec{r}_{j} - \vec{r}_{k} )^{2} /{\text{d}}r^{2} } \right] $$, $$ k = j \pm 1 $$ and $$ \vec{F}_{j}^{ \bot } = K^{ \bot } (2\vec{r}_{j} - \vec{r}_{j + 1} - \vec{r}_{j - 1} ) $$.

**Fig. 5:**
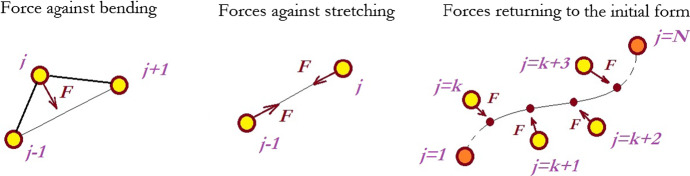
Different forces acting within one filament in a discrete approach. The force acting against bending tends to return an intermediate node to the position between the two nearest ones. The force acting against stretching conserves trial distance between the two nearest neighbors. The force conserving its original form returns the positions of all the internal nodes to their initial places (Color figure online)

Here, a couple of tricks is used to simplify the model further. The longitudinal force, $\vec{F}_{jk}^{||}$, is described by a two-minima potential, which tends to keep a distance between the points $\vec{r}_{j}$ and $\vec{r}_{j \pm 1}$ close to the equilibrium length of the segment $dr$. The transversal force, $\vec{F}_{j}^{ \bot }$, keeps $\vec{r}_{j}$ close to the mean value $(\vec{r}_{j + 1} + \vec{r}_{j - 1} )/2$ between its nearest neighbors and tends to hold the angle between the neighboring segments close to 180° (Fig. 5).

External force, in this case, appears due to the adhesive nature of the setal surface which attracts the ends of the fibers by the combination of molecular and capillary forces. For the sake of simplicity, we simulated this force in this study by the gradient of Morse potential $U_{\text{VdW}} (r) = U_{0} (1 - \exp ( - r/r_{0} ))^{2}$, where $r$ is the distance between the end of fiber and surface. This potential has a form typical for such kind of attraction forces having minimum at characteristic distance from the surface $r_{0}$.

Besides the interaction to the nearest neighbors along the individual fiber, the system has an attraction between different fibers as well. Soft parts of every fiber, which normally are physically thin and flexible, interact with corresponding regions of other fibers of the array. Interaction force has the same (Van der Waals or capillary) origin as their attraction to the hard substrate. Due to this, it is natural to take it in the same form $U_{\text{interact}} (r_{jk} ) = U_{0} (1 - \exp ( - r_{jk} /r_{0} ))^{2}$ with comparable characteristic parameters $U_{0} ,r_{0}$. For simplicity of the model, one can also reduce mutual interaction between the fibers by the interaction of the nearest neighbors: $r_{nn + 1} = \left| {\vec{r}_{n} - \vec{r}_{n \pm 1} } \right|$ along the array. Please, do not mix it with the interaction along an individual fiber. So, we simultaneously have simplification in both “channels”: in interaction along the fibers and between them.

For studied problem, one can neglect effects of inertia and treat the system as an over-damped one. In this approximation, differential equation of motion does not contain the second time derivative and can be formally written in the general form of Eq. (9) $\partial \vec{r}/\partial t = \vec{F}/\gamma$, as above.

The only, but important difference is that here that the model includes physical parameters and interactions. For example, here $\gamma$ is a dissipative constant, and force includes mentioned above interactions $\vec{F} = \vec{F}_{\text{elastic}} + \vec{F}_{\text{VdW}} + \vec{F}_{\text{interact}}$. But, if we normalize $\gamma^{ - 1}$ to a typical relaxation times of the system, the equation of motion is formally reduced to its simplest form $\partial \vec{r}/\partial t = \vec{F}$ and formally written above for a very general case.

**Fig. 6:**
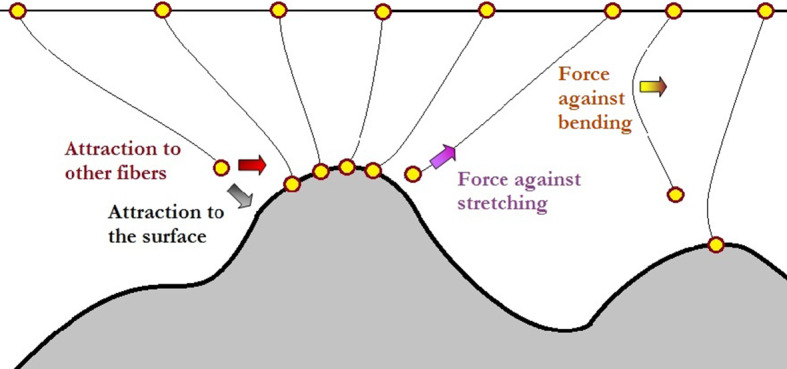
Forces acting in the system of fibers in the presence of substrate. For each fiber, there are at least two external forces (attraction to the surface and to nearest fibers) and two internal forces acting against its deformations (stretching and bending) (Color figure online)

Despite of the apparent simplicity, it includes already large number of interactions, but still needs some further additions. In this particular problem, which is devoted to the role of elasticity gradient, the stiffness of fibers is continuously varied along vertical coordinate. To make this variation definite, we applied analytical smooth step function $\varTheta (y) = 1/[1 + \exp ( - (y - y_{0} )/\Delta )]$ with regulated position of place starting from which the fiber can be easily bent $y_{0}$ and width $\Delta$ of the interval where the elasticity essentially changes. This function tends to one, when $y \ll y_{0}$, and gradually goes to zero in the opposite limit $y \gg y_{0}$. This allows modeling different variants of transversal stiffness dependencies. At the first glance, it seems to be an absolutely trivial modification. However, it is very strong advantage of the discrete approach, especially for modern matrix-based computer calculations (Fig. 6).

The gradient of elasticity is the one solution among many “ideas” which living nature is using to enhance the contact area with the surface. In particular, biological hairy adhesive systems of different animals, involved in locomotion, rely not on flat punches on their tips, but rather on spatulate structures. In principle, experimental observations allow numerous explanations of importance of this particular contact geometry, for example, an enhancement of adaptability to the rough substrate and contact formation by shear force rather than by normal load. It is very difficult to judge about real mechanism just from experimental observations, especially if the subject of the study is the role of spatulate terminal elements in biological fibrillar adhesion.

Fortunately, now we are ready to apply numerical approach to study dynamics of such spatulate tips during contact formation on rough substrates. It is expected that such a model is able to demonstrate definitely that the contact area increases under applied shear force. As before, the numerical model here deals with the problem of optimization. It is expected that applied shear force has an optimum when maximal contact is formed but no slip occurs.

It is important that dynamic model can be constructed in 3D space. It makes possible to study how the contact on a rough substrate can be generated by shear, especially if the spatulae are initially not aligned in the plane of the substrate. Earlier, we used such numerical approach to study the dynamics of spatulate tips during contact formation on rough substrates (Filippov et al. 2011).

Combining different possibilities discussed above, one can quite realistically reproduce the system and answer the questions: What is the role of the thickness (elasticity) gradient on spatulae adhesion? Does applied shear force contribute to the enhancement of contact area on the rough substrate? And of course: Is there any optimal shear distance/force for the single spatula?

The situation here is relatively simple and complex at the same time, and as result, its description involves a number of different features independently discussed above. First of all, we deal with Van der Waals attraction to the surface which competes with the resistance of the spatula to bending. According to the theory of elasticity, the elastic energy of flexible plate in 3D space is given by quite complex integral (Landau and Lifshitz 1976):

10 $$ W_{\text{elastic}} = \frac{E}{{24(1 - \nu^{2} )}}\iint {{\text{d}}x{\text{d}}yh^{3} (x,y)\left\{ {\left( {\frac{{\partial^{2} z}}{{\partial x^{2} }} + \frac{{\partial^{2} z}}{{\partial y^{2} }}} \right)^{2} + 2(1 - \nu )\left[ {\left( {\frac{{\partial^{2} z}}{\partial x\partial y}} \right)^{2} - \frac{{\partial^{2} z}}{{\partial x^{2} }}\frac{{\partial^{2} z}}{{\partial y^{2} }}} \right]} \right\}} $$

where $E$ is the Young’s modulus of the plate material and $\nu$ is the Poisson ratio which is typically equal to $\nu = 1/3$. The competition of the forces leads to the (over-damped) dynamics of the system which is described as before by the equation of motion in the direction normal to the substrate (vertical, z-component):

11 $$ \gamma \frac{\partial z(x,y)}{\partial t} = - \frac{{\delta W_{\text{elastic}} \left[ z \right]}}{\delta z} - \frac{{\delta U_{\text{VdW}} \left[ z \right]}}{\delta z}, $$

Van der Waals interaction also produces force parallel to the substrate (horizontal, *x*-component): $F_{\text{VdW}}^{x} = - \delta U_{\text{VdW}} \left[ {z(x)} \right]/\delta x$. This force competes with an external shear force $F_{{}}^{x}$. When $F_{{}}^{x}$ exceeds the total resistance of all instantly bonded segments $\int {{\text{d}}x{\text{d}}y} F_{\text{VdW}}^{x} > \left| {F^{x} } \right|$, the whole spatula moves along the x-direction according to the equation:

12 $$ \gamma \partial x/\partial t = F^{x} - \int {{\text{d}}x{\text{d}}yF_{\text{VdW}}^{x} } $$

A typical configuration of the moving system can be described as follows. The “spatula” plate is initially attached to the surface by the Van der Waals force $F_{\text{VdW}} = - \frac{{\delta U_{\text{VdW}} \left[ z \right]}}{\delta z}$ by one of its end segments. It then relaxes in the course of time to an equilibrium state, in which it adheres to the surface by additional segments. The rate of attachment depends on the angle $\alpha$ between the plate and surface, normally being faster for smaller angles $\alpha$.

**Fig. 7:**
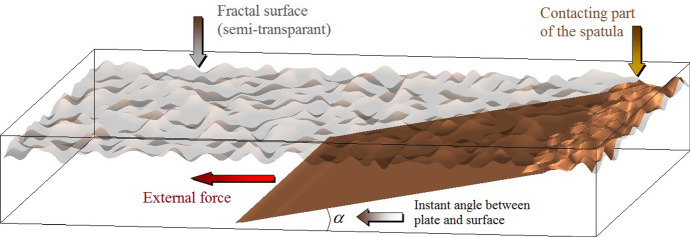
Conceptual scheme of the model representing development of the contact between the single spatula and fractal surface. Being initially attached by its negligible part, but pulled by external horizontal force, the thin two-dimensional plate (spatula) gradually rotates to the smaller angle between it and the surface. With time, this dynamic process favors much better (almost perfect) contact between the spatula and substrate surface (Color figure online)

If the external force is nonzero $F \ne 0$, it pulls the plate to the left and competes with the Van der Waals attachment $F_{\text{VdW}}$ of the “glued” segments. If the total attachment force is stronger than the horizontal component of the external force $\int {{\text{d}}x{\text{d}}y} F_{\text{VdW}}^{x} > \left| {F^{x} } \right|$, the spatula does not slide along the *x*-axis. However, the part which remains unattached can rotate and approach the surface $z(x,y) \to < Z >$ due to the action of the vertical component of the force $F^{z} > 0;F^{z} \sim z$ (Fig. 7).

This rotation reduces distances between hard and flexible surfaces and can greatly enhance total adhesion. Generally, one can expect that a stronger shear force will cause faster attachment. However, if the shear force is too strong, it can exceed the Van der Waals locking and even lead to detachment of previously attached segments. In this case, the plate will start to slip along the surface, and its rotation stops and additional segments do not adhere to the substrate.

These qualitative considerations give rise to the already mentioned optimization problem: Up to which extent one can vary shear force to stimulate attachment without rupturing the contact? To answer this question, we have performed two sets of numerical simulations: with a fixed initial inclination angle $\alpha$ and varying force $F$ as well as with a fixed force and varying angle. Complete results of numerical experiments have been presented in the earlier publication (Filippov et al. 2011). Here, we will comment only main results of general interest.

As expected, the higher force $F$ leads to a faster decrease in the inclination angle. If the force is smaller than some critical value for detachment $F < F_{\text{crit}}$, then the plate gradually tends to a horizontal orientation for $t \to \infty$. When the force approaches the critical value $F_{\text{crit}}$, it becomes capable of breaking some of the already attached bonds and of slight shift of the plate in the horizontal direction. Finally, if the force exceeds the critical value (a threshold) $F_{\text{crit}}$, it completely breaks an initial anchoring and causes permanent sliding of the plate. In this regime, the plate does not rotate and does not further approach the horizontal orientation, thus not leading to any increase in adhesion.

It is natural that in the biological attachment system, the animal cannot control the state (attachment and orientation) of each individual spatula, but presumably can monitor a total resistance force of the entire spatulae array, keeping it close to, but not exceeding the critical shear force value. This is very interesting and non-trivial result from both biological and mathematical points of view. It shows that complex and essentially nonlinear living system can solve an optimization problem without balancing of all the forces in exactly static state, but rather by very slow dynamics at the conditions close to the critical threshold.

## Electronic supplementary material

Below is the link to the electronic supplementary material.

Movie 1.1. Attraction of the population to the “ecological niche.” The niche or “survival coefficient” is represented by the bold black line. Consequent stages of the evolution are shown by the curves with the colors changing from red to blue (MP4 207 kb)

Movie 1.2. Complex trajectory caused by a pursuit in phenotype space. The effective potential is depicted by the color map. (Darker green color corresponds to the attracting minimums of the potential or, as it is explained in the main text, to the maximums of the survival coefficient.) It is seen that depending on the particular configuration and initial conditions, the trajectory can either visit some minima many times or never visit them at all (MPG 2250 kb)

Movie 1.3. Collective motion of the discrete predator–prey system. The predators are attracted to the prey items which try to escape from them and collect in some regions of space causing deeper instant valleys of effective potential (shown by the colored surface). The predators are preferably attracted to these regions, leading to the dispersal of the prey items, etc. (AVI 16346 kb)

Movie 1.4 The effective “adiabatic behaviour” obtained in discrete model, where collective motion of the two groups of predator–prey pairs with essentially different mobilities is studied. It is seen directly that both fast and slow subgroups treat either instant or time-averaged configuration of another group (shown by small and big circles, respectively) as the effective potentials (MPG 2238 kb)
